# Combination of dynapenia and abdominal obesity affects long-term physical performance trajectories in older adults: sex differences

**DOI:** 10.1093/ajcn/nqac023

**Published:** 2022-01-31

**Authors:** Roberta de Oliveira Máximo, Dayane Capra de Oliveira, Paula Camila Ramirez, Mariane Marques Luiz, Aline Fernanda de Souza, Maicon Luís Bicigo Delinocente, Andrew Steptoe, Cesar de Oliveira, Tiago da Silva Alexandre

**Affiliations:** Postgraduate Program in Physical Therapy, Federal University of Sao Carlos, Sao Carlos, Sao Paulo, Brazil; Postgraduate Program in Physical Therapy, Federal University of Sao Carlos, Sao Carlos, Sao Paulo, Brazil; Postgraduate Program in Physical Therapy, Federal University of Sao Carlos, Sao Carlos, Sao Paulo, Brazil; Postgraduate Program in Physical Therapy, Federal University of Sao Carlos, Sao Carlos, Sao Paulo, Brazil; Postgraduate Program in Physical Therapy, Federal University of Sao Carlos, Sao Carlos, Sao Paulo, Brazil; Postgraduate Program in Gerontology, Federal University of Sao Carlos, Sao Carlos, Sao Paulo, Brazil; Department of Epidemiology and Public Health, University College London, London, United Kingdom; Department of Epidemiology and Public Health, University College London, London, United Kingdom; Postgraduate Program in Physical Therapy, Federal University of Sao Carlos, Sao Carlos, Sao Paulo, Brazil; Postgraduate Program in Gerontology, Federal University of Sao Carlos, Sao Carlos, Sao Paulo, Brazil; Department of Epidemiology and Public Health, University College London, London, United Kingdom; Gerontology Department, Federal University of Sao Carlos, Sao Carlos, Sao Paulo, Brazil

**Keywords:** waist circumference, grip strength, SPPB, physical performance, trajectories

## Abstract

**Background:**

There is little epidemiological evidence of sex differences in the association between dynapenic abdominal obesity and the decline in physical performance in older adults.

**Objectives:**

The aims of the present study were to investigate whether the decline in physical performance is worse in individuals with dynapenic abdominal obesity and whether there are sex differences in this association.

**Methods:**

Of 6183 individuals aged ≥60 y from the English Longitudinal Study of Ageing, 2308 participants with missing data were excluded. Therefore, a longitudinal analysis was conducted with 3875 older adults. Abdominal obesity was determined based on waist circumference (>102 cm for males, and >88 cm for females), and dynapenia was based on grip strength (<26 kg for males, <16 kg for female). The sample was divided into 4 groups: nondynapenic/nonabdominal obesity (ND/NAO), nondynapenic/abdominal obesity (ND/AO), dynapenic/nonabdominal obesity (D/NAO), and dynapenic/abdominal obesity (D/AO). Decline in physical performance in an 8-y follow-up period was analyzed using generalized linear mixed models.

**Results:**

At baseline, both male (−1.11 points; 95% CI: −1.58, −0.65 points; *P* < 0.001) and female (−1.39 points; 95% CI: −1.76, −1.02 points; *P* < 0.001) with D/AO had worse performances on the Short Physical Performance Battery (SPPB) than their counterparts in the ND/NAO group. Over the 8-y follow-up, males with D/AO had a faster rate of decline in the SPPB performance compared with males in the ND/NAO group (−0.11 points/y; 95% CI: −0.21, −0.01 points; *P* = 0.03).

**Conclusions:**

D/AO is associated with a stronger decline in physical performance in males but not in females. The identification and management of dynapenic abdominal obesity could be essential to avoiding the first signs of functional impairment in older males.

## Graphical Abstract

**Graphical Abstract:**
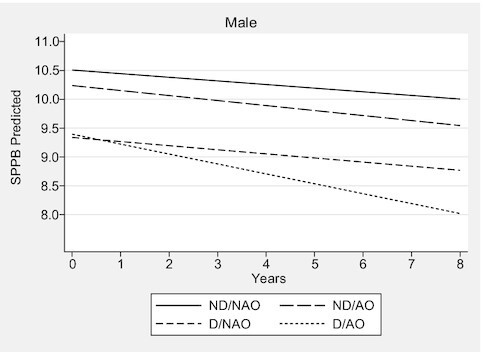

## Introduction

The decline in physical performance is commonly the first indicator of impaired function in older adults (1) and is considered a preclinical transition phase toward disability (2, 3), predisposing these individuals to a greater risk of negative outcomes (1). Therefore, screening for this decline has been widely promoted in the clinical geriatric setting (4).

Age-related changes in motor neuron function and muscle contractile properties lead to the loss of muscle strength, which is known as dynapenia (5). This process is faster in males despite their greater muscle mass and strength throughout life compared with females (6–8). Furthermore, fat distribution shifts from subcutaneous to abdominal deposits with aging, whereas fat mass tends to decrease or remain stable (9–12). This distribution occurs earlier in males than in females due to the menopause (12, 13). The accumulation of abdominal fat exacerbates dynapenia (9, 14–17), especially in males (17). This process is mediated by low-grade inflammation, which promotes insulin resistance and muscle catabolism and affects the repair of motor neurons (18, 19). In this way, dynapenic abdominal obesity (a combination of dynapenia and abdominal obesity) could exert an impact on physical functioning differently between males and females.

Dynapenic abdominal obesity was related to gait speed decline over an 8-y follow-up in a previous study conducted by de Oliveira Máximo et al. (20) with 2294 individuals aged ≥60 y free of mobility limitation at baseline. However, conflicting results are found when considering dynapenic obesity defined by the combination of dynapenia and general obesity. For example, exploring the cross-sectional relation in older adults, Bouchard and Janssen (21) and Yang et al. (22) found that dynapenic obesity was associated with low gait speed. In a longitudinal study, on the other hand, Batsis et al. (23) found no decline in gait speed in individuals with dynapenic obesity over the 4-y follow-up period.

The decline in physical performance is considered a component that precedes the onset of disability (2). Moreover, consistent evidence shows that dynapenic abdominal obesity is associated with disability regarding basic (24) and instrumental (25) activities of daily living. Therefore, the association between dynapenic abdominal obesity and the decline in physical performance needs to be investigated. For such, measures that incorporate a broader spectrum of functioning would be useful, such as the Short Physical Performance Battery (SPPB), which measures gait speed as well as balance, lower limb strength, and endurance.

Therefore, the aims of the present study were to investigate whether the decline in physical performance is worse in individuals with dynapenic abdominal obesity and whether there are sex differences in this association. Our hypothesis is that the decline in physical performance is worse in individuals with dynapenic abdominal obesity compared with those with dynapenia or abdominal obesity alone and nondynapenic/nonabdominal obesity, and that males with dynapenic abdominal obesity have a worse physical performance than females.

## Methods

### Study population

The data used in this study were from the English Longitudinal Study of Ageing (ELSA), which is an ongoing panel study involving community-dwelling individuals in England aged ≥50 y. ELSA began in 2002, and the sample was composed of participants of the Health Survey for England (HSE), which involved a nationally representative sample selected using a multistage stratified probability sampling design (26). Follow-up interviews in ELSA occur every 2 y, and health examinations are performed by a nurse every 4 y. The first health examination occurred in 2004–2005. A detailed description of the study can be found in a previous publication (27).

The sample of the present study comprised 6183 individuals aged ≥60 y in 2004, when anthropometric and physical performance data were collected for the first time. Of these individuals, 2308 were excluded due to missing data on the SPPB, grip strength, waist circumference, or other covariates, resulting in a final sample of 3875 individuals at baseline (**Supplemental Figure 1**). These measures were not obtained for individuals who were incapable of: *1*) performing the walk tests without the use of a gait-assistance device; *2*) standing up from a chair a single time without using the arms; *3*) performing the standing balance tests; *4*) performing the grip strength test; or *5*) remaining in the standing position for the measurement of waist circumference. The participants were reevaluated after 4 y (2008) and 8 y (2012).

### Ethical approval and informed consent

Ethical approval and experimental protocols for ELSA were granted by the Multi-Centre Research and Ethics Committee (MREC/01/2/91). Respondents in ELSA gave their informed consent to participate in the study. The authors confirm that all research and methods were performed in accordance with approved guidelines and regulations.

### Physical performance assessment

The SPPB is used to determine the physical performance of older adults through the combined assessment of static balance (feet side by side, semitandem, and tandem), the 2.4-m walk test, and repeated chair stands (28). Each physical performance measure was categorized from 0 to 4 points, with 0 corresponding to the inability to perform the test and 4 corresponding to the highest level of performance. The complete battery ranges from 0 to 12 points, with higher scores denoting a better physical performance (28). In the present study, the outcome (SPPB score) was treated as a discrete variable.

### Anthropometric measures and classification of groups

Grip strength was measured using a handgrip dynamometer (Smedley; range: 0 to 100 kg). During the test, the participant remained standing with the arm alongside the trunk and the elbow flexed at 90 degrees (29). Three maximum strength trials were performed with a 1-min rest period between readings, and the highest value was considered for the analysis. Dynapenia was defined as grip strength <26 kg for males, and <16 kg for females (30).

Waist circumference was measured using a metric tape at the midpoint between the lowest rib and the upper edge of the iliac crest. The measurement was made twice at the end of the expiratory phase of the respiratory cycle (29). A third measurement was performed if the difference between the first 2 measurements was >3 cm. Abdominal obesity was defined as waist circumference >102 cm for males, and >88 cm for females (31, 32).

Four time-varying groups were created based on the ab-sence/presence of abdominal obesity and dynapenia: nondy-napenic/nonabdominal obesity (ND/NAO); nondynapenic/abdominal obesity (ND/AO); dynapenic/nonabdominal obesity (D/NAO); and dynapenic/abdominal obesity (D/AO) (25).

### Covariates

The socioeconomic variables were: age grouped into three 10-y categories; marital status (married compared with not married); total household wealth (quintiles); and level of education. The English 3-way education system was qualified to a level lower than “O-level” or equivalent (0–11 y of schooling), a level lower than “A-level” or equivalent (12–13 y), and a higher qualification (>13 y) (25, 33).

Smoking was determined by asking the participants whether they were nonsmokers, ex-smokers, or current smokers. Regarding alcohol intake, the participants were classified as nondrinkers or rare drinkers (up to once per week), frequent drinkers (2–6 times per week), or daily drinkers (24). Physical activity level was determined using an instrument validated by the HSE (34), which considers the frequency of participation in vigorous, moderate, and mild physical activities (more than once per week, once per week, 1–3 times per month, or almost never). Lifestyle was classified as sedentary (no weekly physical activity) or active (mild, moderate, or vigorous physical activity at least once per week) (25).

Health status was ascertained by self-reported medical diagnosis of diabetes, hypertension, stroke, heart disease, lung disease, cancer, osteoarthritis, osteoporosis, and number of falls in the previous 12 months. Pain was assessed by asking the participants whether they were often troubled by pain in the hips, knees, or feet when walking; this variable was dichotomized as no pain or pain (any degree) (35). Cognitive function was evaluated based on the global score of the immediate and delayed recall test (range: 0 to 20 words) (36). Depressive symptoms were determined using the Center for Epidemiologic Studies Depression Scale (CES-D), considering a cutoff of ≥4 points (37).

Weight (kilograms) was measured using a Tanita electronic scale with the participant barefoot and wearing light clothing. Height (meters) was measured using a standardized Leicester portable stadiometer. BMI was calculated by dividing weight in kilograms by height in meters squared (kg/m^2^). BMI ≥30 kg/m^2^ was considered indicative of obesity. Weight change was assessed by comparing baseline weight (kilograms) to weight at 4-y and 8-y follow-up evaluations, because weight loss can affect the association between abdominal obesity and the decline in muscle strength:

(1) \begin{eqnarray*} && \left[({\rm{weight}}\,{\rm{at}}\,4 - {\rm{y}}\,{\rm{follow}} - {\rm{up}} - {\rm{weight}}\,{\rm{at}}\,{\rm{baseline}})/\right. \\ &&\qquad \left. {\rm{weight}}\,{\rm{at}}\,{\rm{baseline}} \times 100\right] \end{eqnarray*}

and

(2) \begin{eqnarray*} && \left[({\rm{weight}}\,{\rm{at}}\,8 - {\rm{y}}\,{\rm{follow}} - {\rm{up}} - {\rm{weight}}\,{\rm{at}}\,{\rm{baseline}})/\right. \\ &&\qquad \left. {\rm{weight}}\,{\rm{at}}\,{\rm{baseline}} \times 100\right] \end{eqnarray*}

In comparison with weight at baseline, the individuals were categorized as having stable weight, weight loss ≥5%, and weight gain ≥5% over follow-up (38).

### Statistical analysis

Differences in baseline characteristics between *1*) included individuals and those excluded due to missing data on the SPPB, grip strength, waist circumference, or other covariates; and *2*) the 4 groups classified according to abdominal obesity and dynapenia status were evaluated using the χ^2^ test, ANOVA, and Tukey post hoc test. For all analyses, a *P* value <0.05 was considered indicative of statistical significance.

Generalized linear mixed models (GLMMs) stratified by sex were performed to estimate the trajectories of physical performance as a function of abdominal obesity and dynapenia status. We assumed normal distribution of the outcome, and the XTMIXED procedure was used with an identity link and covariance structure maximum-likelihood estimates (mle) in Stata 14 SE (StataCorp) (39). GLMMs were chosen because such models are more appropriate for unbalanced data from studies with repeated measures and enable the statistical modeling of time-dependent changes in the outcome variable (SPPB) and in the magnitude of associations between variables (40, 41). A full-model approach (42) was used with adjustment for a wide range of potential covariates defined a priori as being associated with the decline in physical performance (43). All covariates were treated as time-varying (i.e., when a variable changes over time for the subjects) (44).

In the GLMMs, the intercept represents differences in the mean SPPB score between the ND/AO, D/NAO, and D/AO groups and the reference group (ND/NAO) at baseline. The coefficient for time represents SPPB performance decline in the reference group. Lastly, the coefficient for the interaction between time and ND/AO, D/NAO, and D/AO represents differences in slope (the annual rate of decline in SPPB performance) between each of the 3 groups and the reference group. The results were reported as β coefficients and 95% CIs.

Three sensitivity analyses were performed. The first was to investigate whether abdominal obesity (yes/no) and dynapenia (yes/no), when analyzed separately, would be capable of modifying the associations found in the original models. The second was to investigate whether dynapenic obesity, defined using BMI ≥30 kg/m^2^ rather than abdominal obesity, is associated with decline in physical performance. The third was to investigate whether the association between dynapenic abdominal obesity and decline in physical performance is modified when excluding individuals with a low SPPB score at baseline (≤8 points). Moreover, statistics to estimate average population parameters, such as the marginal average, were used from predictions of a previously fitted model.

## Results

Of the 3875 participants at baseline, 2932 and 2436 were reevaluated at the 4-y and 8-y follow-ups, respectively. Slightly more than 62.9% of the initial sample participated in the 3 waves, and 75.7% participated in 2 waves of the study. The baseline characteristics according to abdominal obesity and dynapenia status stratified by sex are displayed in **Table 1**.

**TABLE 1:** Baseline characteristics of males and females from the ELSA study (2004) according to abdominal obesity and dynapenia status^1^

	Males				Females			
	ND/NAO	ND/AO	D/NAO	D/AO	ND/NAO	ND/AO	D/NAO	D/AO
	*n* = 933	*n* = 753	*n* = 59	*n* = 35	*n* = 881	*n* = 1054	*n* = 82	*n* = 78
Age, y	69.5 ± 6.9	69.5 ± 6.5	79.3 ± 6.5^a,b^	75.9 ± 9.5^a,b^	69.5 ± 7.4	69.5 ± 6.6	77.8 ± 7.2^a,b^	74.2 ± 8.0^a,b,c^
60–69, %	55.4	52.2	8.4^a,b^	31.4^a,b^	57.5	54.5	14.6^a,b^	29.5^a,b^
70–79, %	35.2	39.6	42.4	31.5	30.1	37.5^a^	37.8	42.3
≥80, %	9.4	8.2	49.2^a,b^	37.1^a,b^	12.4	8.0^a^	47.6^a,b^	28.2^a,b^
Marital status (not married), %	22.0	20.6	40.7^a,b^	31.4	40.7*	39.7*	69.5^*,a,b^	57.7^a,b^
Total household wealth in quintiles, %								
First (highest)	29.6	23.2^a^	15.3	2.9^a,b^	27.2	19.1^a^	12.2^a^	10.3^a^
Second	24.0	21.1	25.4	25.7	23.4	21.3	17.1	17.9
Third	21.5	21.5	15.3	17.1	18.8	21.8	20.7	26.9
Fourth	14.3	19.8^a^	23.7	28.6	16.8	19.9	23.2	30.8^a^
Fifth (lowest)	9.7	13.1	20.3^a^	22.9^a^	12.6	16.7	26.8^a^	14.1
Not reported, %	0.9	1.3	0.0	2.8	1.2	1.2	0.0	0.0
Level of education, %								
Higher qualification	33.4	27.7	18.6	5.7^a,b^	21.8*	16.2^*,a^	8.5^a^	9.0^a^
Level lower than “A level” or equivalent	23.8	21.0	13.6	17.1	23.3	19.9	15.9	17.9
Level lower than “O level” or equivalent	42.8	51.3^a^	67.8^a^	77.2^a,b^	54.9*	63.9^*,a^	75.6^a^	73.1^a^
Smoking, %								
Nonsmoker	30.6	24.4^a^	15.2^a^	17.1	47.2*	44.7*	50.0*	42.3
Ex-smoker	56.4	64.8	67.8^a^	74.3	41.1*	45.1*	40.2*	48.7
Smoker	13.0	10.8	17.0	8.6	11.7	10.2	9.8	9.0
Alcohol intake, %								
Nondrinker or rare drinker	10.1	11.5	8.5	5.7^b^	20.0*	23.9*	28.0*	30.8*
Frequent drinker	39.6	42.0	44.1	45.7	43.5	43.9	39.0	39.7
Daily drinker	42.3	38.5	23.7^a^	22.9	30.5*	23.6*	20.8	21.8
Did not answer	8.0	8.0	23.7^a,b^	25.7^a,b^	6.0	8.6	12.2	7.7
Sedentary behavior, %	2.5	2.1	5.1	2.9	1.7	2.9	7.3^a^	7.7^a^
Hypertension, %	36.3	52.6^a^	42.4	54.3	37.9	55.1^a^	45.1	64.1^a^
Diabetes, %	7.9	11.8	11.9	25.7^a^	2.3*	9.9^a^	4.9	14.1^a^
Cancer, %	7.1	9.8	8.5	8.6	9.8	9.6	13.4	9.0
Lung disease, %	14.7	16.6	13.6	28.6	16.8	20.5	17.1	24.4
Heart disease, %	25.8	26.0	32.2	34.3	20.5	20.9	28.0	28.2
Stroke, %	4.9	4.4	5.1	2.9	3.6	3.2	9.8^b^	5.1
Osteoarthritis, %	22.7	32.1^a^	44.1^a^	51.4^a^	36.5*	45.5^*,a^	72.0^*,a,b^	75.6^a,b^
Osteoporosis, %	1.2	1.7	3.4	8.6	13.1*	9.7*	15.9	15.4
Joint pain, %	15.8	22.4^a^	25.4	31.4^a,b^	21.0	30.9^a^	32.9	48.7
Number of falls in previous 12 months (mean ± SD)	0.4 ± 1.27	0.5 ± 2.28	0.7 ± 1.20	1.0 ± 3.44	0.6 ± 1.94	0.7 ± 1.98	0.8 ± 1.30	1.1 ± 2.06
Depressive symptoms, %	6.8	7.8	10.2	11.4	11.1*	15.5^*a^	24.4^a^	16.7
Memory score, points (mean ± SD)	9.5 ± 3.2	9.3 ± 3.2	6.8 ± 3.4^a,b^	8.3 ± 3.7	10.3 ± 3.5*	10.0 ± 3.3*	8.0 ± 4.1^a,b^	8.7 ± 3.7^a,b^
Grip strength, kg (mean ± SD)	39.6 ± 7.4	40.6 ± 7.5^a^	21.0 ± 4.8^a,b^	22.5 ± 2.9^a,b^	24.4 ± 4.9*	24.7 ± 5.0*	12.8 ± 2.2^*,a,b^	13.1 ± 2.6^*,a,b^
Waist circumference, cm (mean ± SD)	93.7 ± 6.2	110.3 ± 7.0^a^	92.5 ± 7.0^b^	109.6 ± 5.6^a,c^	80.2 ± 5.6*	98.5 ± 8.3^*,a^	79.4 ± 6.1^*,b^	97.9 ± 8.2^*,a,c^
Height, m (mean ± SD)	1.71 ± 0.7	1.71 ± 0.6^a^	1.65 ± 0.6^a,b^	1.65 ± 0.6^a,b^	1.60 ± 0.6*	1.60 ± 0.6^*,a,b^	1.60 ± 0.7^*,a,b^	1.50 ± 0.6^*,a,b^
BMI ≥30 kg/m^2^, %	1.9	51.7^a^	0.0	42.9^a^	1.6	51.4^a^	0.0	47.4^a^

1 χ^2^ test performed for categorical variables; ANOVA and Tukey post hoc test performed for continuous variables to evaluate differences in baseline characteristics of 4 groups classified according to abdominal obesity and dynapenia status. Data expressed as percentage or mean ± SD. Statistical significance *P* < 0.05. Males, *n* = 1780; females, *n* = 2095. D/AO, dynapenic/abdominal obesity; D/NAO, dynapenic/nonabdominal obesity; ELSA, English Longitudinal Study of Ageing; ND/AO, nondynapenic/abdominal obesity; ND/NAO, nondynapenic/nonabdominal obesity. *Significant sex difference in each group. ^a^Significantly different from ND/NAO in each sex. ^b^Significantly different from ND/AO in each sex. ^c^Significantly different from D/NAO in each sex.

At baseline, the prevalence of D/AO and ND/AO was slightly higher in females than males [3.7% (95% CI: 3.0, 4.6%) compared with 2.0% (95% CI: 1.4, 2.7%), and 50.3% (95% CI: 48.2, 52.5%) compared with 42.3% (95% CI: 40.0, 44.6%), respectively]. No difference in the prevalence of D/NAO was found between sexes [3.3% (95% CI: 2.6, 4.3%) compared with 3.9% (95% CI: 3.2, 4.8%)].

In the analysis comparing included and excluded individuals due to missing data, excluded individuals were mainly female, older, and not married, had less schooling and income, smoked more, had a lower memory score, lower handgrip strength, higher waist circumference, higher BMI as well as more falls, sedentary behavior, depressive symptoms, and greater frequencies of lung disease, heart disease, diabetes, hypertension, stroke, osteoarthritis, and osteoporosis (see **Supplemental Table 1**).

**Table 2** shows the estimated parameters of the GLMMs for the change in SPPB as a function of abdominal obesity and dynapenia status per sex in the 8 y of follow-up. The group with ND/NAO (both sexes) underwent significant decline in performance on the SPPB over time. At baseline, both males (−1.11 points; 95% CI: −1.58, −0.65 points; *P* < 0.001) and females (−1.39 points; 95% CI: −1.76, −1.02 points; *P* < 0.001) with D/AO had worse performances on the SPPB than their counterparts in the ND/NAO group.

**TABLE 2:** Generalized linear mixed model estimated for SPPB scores as a function of abdominal obesity and dynapenia status over 8 y of follow-up in older English males and females^1^

	Males		Females	
	Estimated parameters β (95% CI)		Estimated parameters β (95% CI)	
Time, y	−0.19	(−0.28, −0.10)**	−0.13	(−0.22, −0.04)*
Intercept main effect				
ND/NAO	10.50^†^		10.02^†^	
ND/AO	−0.27	(−0.41, −0.13)**	−0.37	(−0.52, −0.22)**
D/NAO	−1.17	(−1.55, −0.79)**	−0.83	(−1.21, −0.46)**
D/AO	−1.11	(−1.58, −0.65)**	−1.39	(−1.76, −1.02)**
Slope interaction effect				
Time × ND/NAO	10.44^†^		9.97^†^	
Time × ND/AO	−0.02	(−0.06, 0.02)	−0.01	(−0.05, 0.03)
Time × D/NAO	−0.01	(−0.10, 0.08)	0.01	(−0.07, 0.09)
Time × D/AO	−0.11	(−0.21, −0.01)*	0.01	(−0.06, 0.09)

1 Generalized linear mixed models performed to estimate β coefficients and 95% CIs for SPPB scores as a function of abdominal obesity and dynapenia status in older adults. Model adjusted for age, total household wealth, years of schooling, marital status, smoking status, sedentary behavior, hypertension, diabetes, lung disease, heart disease, stroke, osteoarthritis, osteoporosis, number of falls, joint pain, depressive symptoms, mean memory score, and change in weight. Intercept represents differences in mean SPPB score between ND/AO, D/NAO, and D/AO and the reference group (ND/NAO) at baseline. Time represents SPPB performance decline in reference group. Slope represents estimated changes in SPPB scores per unit of time between group in question and reference. ^*,**^Significantly different from ND/NAO: *P < 0.05, **P < 0.001. ^†^Indicates margins to reference group. Males, *n* = 1780; females, *n* = 2095. D/AO, dynapenic/abdominal obesity; D/NAO, dynapenic/nonabdominal obesity; ND/AO, nondynapenic/abdominal obesity; ND/NAO, nondynapenic/nonabdominal obesity; SPPB, Short Physical Performance Battery.

**FIGURE 1:**
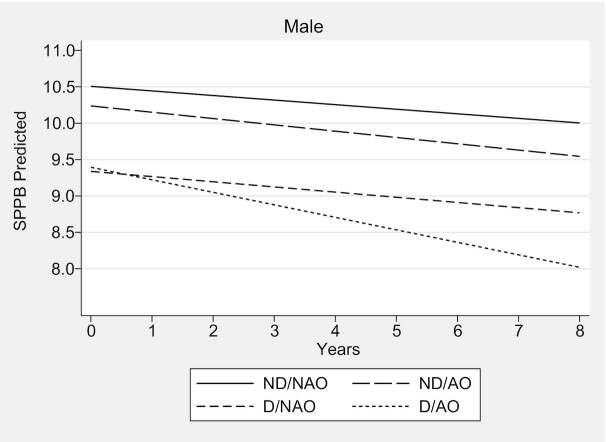
Trajectories of performance on SPPB for males according to abdominal obesity and dynapenia status—the ELSA Study 2004–2012. Predictions for a 60-y-old male, total household wealth = first quintile, higher qualification, married, non-smoker, active, without hypertension, without diabetes, without lung disease, without heart disease, without stroke, without osteoarthritis, without osteoporosis, without falls, without joint pain, CES-D <4 points, mean memory score = 20, and stable weight. Over the 8-y follow-up, only males with D/AO had a faster rate of decline in the SPPB performance compared with males in the ND/NAO group (−0.11 points/y; 95% CI: −0.21, −0.01 points/y; *P* = 0.03); *n* = 1780. CES-D, Center for Epidemiologic Studies Depression Scale; D/AO, dynapenic/abdominal obesity; D/NAO, dynapenic/nonabdominal obesity; ELSA, English Longitudinal Study of Ageing; ND/AO, nondynapenic/abdominal obesity; ND/NAO, nondynapenic/nonabdominal obesity; SPPB, Short Physical Performance Battery.

Over the 8-y follow-up, males with D/AO had a faster rate of decline in SPPB performance compared with males in the ND/NAO group. The estimated parameter for the difference in slope between the 2 groups was −0.11 points/y (95% CI: −0.21, −0.01 points/y; *P* = 0.03) when all other covariates in the model were at zero or at average values, that is, 60 y of age, total household wealth = first quintile, higher qualification, married, nonsmoker, active, without hypertension, without diabetes, without lung disease, without heart disease, without stroke, without osteoarthritis, without osteoporosis, without falls, without joint pain, CES-D <4 points, mean memory score = 20, and stable weight (**Figure 1**, Table 2, **Supplemental Table 2**).

**FIGURE 2:**
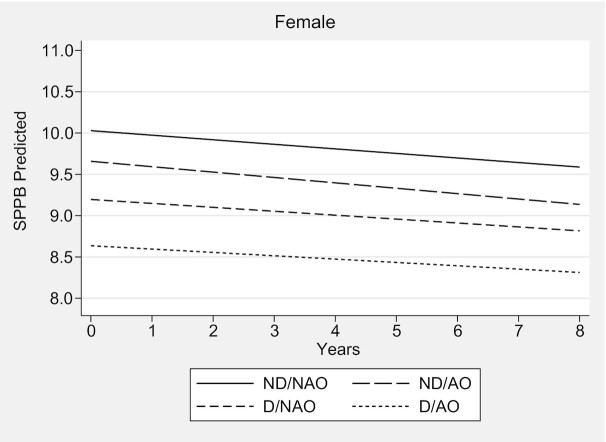
Trajectories of performance on SPPB for females according to abdominal obesity and dynapenia status—the ELSA Study 2004–2012. Predictions for a 60-y-old female, total household wealth = first quintile, higher qualification, married, nonsmoker, active, without hypertension, without diabetes, without lung disease, without heart disease, without stroke, without osteoarthritis, without osteoporosis, without falls, without joint pain, CES-D <4 points, mean memory score = 20, and stable weight. Females with D/AO underwent no significant decline in the performance on the SPPB over time (0.01 points/y; 95% CI: −0.06, 0.09 points/y; *P* > 0.71); *n* = 2095. CES-D, Center for Epidemiologic Studies Depression Scale; D/AO, dynapenic/abdominal obesity; D/NAO, dynapenic/nonabdominal obesity; ELSA, English Longitudinal Study of Ageing; ND/AO, nondynapenic/abdominal obesity; ND/NAO, nondynapenic/nonabdominal obesity; SPPB, Short Physical Performance Battery.

In clinical terms, males and females with D/AO had lower mean SPPB scores at baseline compared with their counterparts in the ND/NAO group (9.39 compared with 10.50 for males, and 8.63 compared with 10.02 for females). However, males with D/AO exhibited a faster decline in the SPPB score at the end of the 8-y follow-up (−1.37 points) (Figure 1, **Supplemental Table 3**), which is considered meaningful change (45). Females with D/AO had a mean decline in the SPPB score of 0.32 points in the same follow-up period (**Figure 2**, Supplemental Table 3).

The first sensitivity analysis considering abdominal obesity and dynapenia as independent conditions showed significant intercept results. However, neither of the 2 conditions alone was associated with a greater SPPB decline based on their slope estimates (**Table 3**). This highlights the importance of the analytical approach adopted in the present study (considering the influence of combinations of abdominal obesity and dynapenia on the long-term decline in physical performance in older adults).

**TABLE 3:** Generalized linear mixed model estimated for SPPB scores as a function of abdominal obesity and dynapenia analyzed as independent conditions over 8 y of follow-up in older English males and females—sensitivity analysis^1^

	Males		Females	
	Estimated parameters β (95% CI)		Estimated parameters β (95% CI)	
Time, y	−0.19	(−0.28, −0.09)**	−0.13	(−0.22, −0.04)*
Intercept main effect				
Without abdominal obesity	10.41^†^		9.93^†^	
Abdominal obesity	−0.25	(−0.39, −0.11)**	−0.39	(−0.53, −0.24)**
Without dynapenia	10.38^†^		9.81^†^	
Dynapenia	−1.04	(−1.34, −0.74)**	−0.93	(−1.20, −0.66)**
Slope interaction effect				
Time × without abdominal obesity	10.34^†^		9.88^†^	
Time × abdominal obesity	−0.03	(−0.07, 0.01)	−0.01	(−0.05, 0.03)
Time × without dynapenia	10.30^†^		9.75^†^	
Time × dynapenia	−0.04	(−0.11, 0.03)	0.02	(−0.04, 0.07)

1 Generalized linear mixed models performed to estimate β coefficients and 95% CIs for SPPB scores as a function of abdominal obesity and dynapenia analyzed as independent conditions in older adults. Model adjusted for age, total household wealth, years of schooling, marital status, smoking status, sedentary behavior, hypertension, diabetes, lung disease, heart disease, stroke, osteoarthritis, osteoporosis, number of falls, joint pain, depressive symptoms, mean memory score, and change in weight. Intercept represents differences in mean SPPB score between abdominal obesity or dynapenia and reference group (without abdominal obesity or without dynapenia) at baseline. Time represents SPPB performance declines in reference group. Slope represents estimated changes in SPPB scores per unit of time between group in question and reference. ^*,**^Significantly different from without abdominal obesity or without dynapenia: *P < 0.05, **P < 0.001. ^†^Indicates margins to reference group. Males, *n* = 1780; females, *n* = 2095. SPPB, Short Physical Performance Battery.

The second sensitivity analysis confirmed that dynapenic obesity (BMI ≥30 kg/m^2^ and grip strength <26 kg for males and <16 kg for female) was not associated with SPPB decline over time (**Table 4**). The third sensitivity analysis, which excluded individuals with a low SPPB score (≤8 points) at baseline, demonstrated that males with D/AO had higher rates of decline in SPPB performance than those in the ND/NAO group (**Supplemental Figures 2** and **3, Supplemental Table 4**).

**TABLE 4:** Generalized linear mixed model estimated for SPPB scores as a function of obesity (BMI ≥30 kg/m^2^) and dynapenia status over 8-y follow-up in older English males and females—sensitivity analysis^1^

	Males		Females	
	Estimated parameters β (95% CI)		Estimated parameters β (95% CI)	
Time, y	−0.17	(−0.26, −0.08)**	−0.15	(−0.25, −0.06)**
Intercept main effect				
ND/NO	10.50^†^		10.08^†^	
ND/O	−0.11	(−0.31, 0.08)	−0.29	(−0.48, −0.10)*
D/NO	−1.08	(−1.41, −0.75)**	−0.98	(−1.29, −0.68)**
D/O	−0.95	(−1.64, −0.27)*	−1.13	(−1.66, −0.60)**
Slope interaction effect				
Time × ND/NO	10.42^†^		9.99^†^	
Time × ND/O	0.02	(−0.03, 0.08)	0.01	(−0.04, 0.06)
Time × D/NO	−0.03	(−0.11, 0.04)	0.02	(−0.05, 0.08)
Time × D/O	−0.08	(−0.22, 0.07)	0.03	(−0.07, 0.13)

1 Generalized linear mixed models performed to estimate β coefficients and 95% CIs for SPPB scores as a function of obesity (BMI ≥30 kg/m^2^) and dynapenia status in older adults. Model adjusted for age, total household wealth, years of schooling, marital status, smoking status, sedentary behavior, hypertension, diabetes, lung disease, heart disease, stroke, osteoarthritis, osteoporosis, number of falls, joint pain, depressive symptoms, mean memory score, and waist circumference. Intercept represents differences in mean SPPB score between the ND/O, D/NO, and D/O and reference group (ND/NO) at baseline. Time represents SPPB performance decline in reference group. Slope represents estimated changes in SPPB scores per unit of time between group in question and reference. ^*,**^Significantly different from ND/NO: *P < 0.05, **P < 0.001. ^†^Indicates margins to reference group. Males, *n* = 1780; females, *n* = 2095. D/NO, dynapenia/nonobesity; D/O, dynapenia/obesity; ND/NO, nondynapenia/nonobesity; ND/O, nondynapenia/obesity; SPPB, Short Physical Performance Battery.

## Discussion

In this large, nationally representative cohort, we demonstrated that older English males with dynapenic abdominal obesity have a stronger decline in physical performance. Moreover, when abdominal obesity and dynapenia were analyzed as independent conditions or when dynapenic obesity was defined by BMI ≥30 kg/m^2^, neither was associated with a stronger decline in physical performance in either males or females, which highlights the importance of dynapenic abdominal obesity as a clinical condition.

Previous studies offer divergent findings regarding the association between dynapenic obesity and poorer physical performance in older adults. Cross-sectional studies conducted by Yang et al. (22) involving 616 males and females aged ≥60 y (BMI ≥25 kg/m^2^ and grip strength) and Bouchard and Janssen (21) involving 2039 individuals aged ≥55 y (body fat mass and leg extensor strength) reported similar results, because individuals with dynapenic obesity had lower gait speed than those without either condition. In a 4-y follow-up study of 2025 individuals aged ≥60 y, on the other hand, Batsis et al. (23) found that the association between dynapenic obesity (BMI ≥30 kg/m^2^ and knee extensor strength) and low gait speed at baseline in both sexes was not confirmed over time.

The most likely reason for the differences found between cross-sectional (21, 22) and longitudinal (23) analyses seems to reside in how obesity is measured. General obesity indicators, such as BMI, are directly related to muscle strength in older people (14, 17) and might not capture age-related changes in body fat distribution over time or differences between the sexes (12, 14, 46, 47). Considering data for 8441 participants aged ≥48 y from the European Prospective Investigation into Cancer–Norfolk, Keevil et al. (14) found that each 4.0-kg/m^2^ increase in BMI corresponded to a 4.28-kg and 1.26-kg increase in grip strength in males and females, respectively, whereas each 10-cm increase in waist circumference corresponded to a 3.56-kg and 1.00-kg reduction in grip strength in males and females, respectively. Moreover, analyzing data for 5181 older participants of the ELSA, de Carvalho et al. (17) found that abdominal obesity was a risk factor for decline in grip strength trajectories in males but not in females in an 8-y follow-up period. Therefore, waist circumference seems to be more appropriate for this assessment in older adults, despite not being as accurate.

The decline in physical performance in older adults is complex and not fully understood. However, the accumulation of abdominal fat and the consequent low-grade inflammation has been associated with the stimulation of processes that exert a negative impact on muscle metabolism (18, 19) and the repair of neurons (5). Thus, individuals with D/AO could experience faster decline in physical performance compared with ND/NAO, ND/AO, and D/NAO groups.

The sex differences found in the present study can be explained by different age-related patterns of body fat distribution and muscle strength decline between males and females. Males exhibit more age-related loss of muscle strength (6, 7) and accumulate abdominal fat earlier, with greater intensity, and with a predisposition toward visceral fat deposition (12, 13). Abdominal obesity is associated with a decline in a variety of neural and hormonal trophic aspects in muscles, given the link to chronic inflammation and the reduction in tolerance to glucose (18, 19). Thus, evidence of the association between abdominal obesity and the exacerbation of the process of dynapenia exclusively in males (17) lends support to the stronger decline in physical performance in males with dynapenic abdominal obesity. In contrast, the buildup of central fat arises at an older age and in a subtler manner in female, occurring after menopause and with subcutaneous deposition (12, 13). Thus, the milder production of inflammatory cytokines due to this alternate fat deposition (48) could attenuate the association between dynapenic abdominal obesity and the decline in physical performance in females over time.

The fact that both males and females with D/AO began the study with worse SPPB scores compared with their counterparts in the ND/NAO group highlights the importance of dynapenic abdominal obesity as a clinical condition that affects physical performance. However, the lack of an association between D/AO and a poorer SPPB performance in females over time might reflect the smaller effect of abdominal fat on the loss of muscle strength, which was milder in females than males with D/AO (*P* < 0.01, data not shown).

The sensitivity analyses showed that not combining abdominal obesity with dynapenia can lead to important associations between these conditions and the decline in physical performance being overlooked, because abdominal obesity and dynapenia alone were not associated with SPPB decline over time (Table 3). This highlights the importance of the analytical approach adopted in the present study. The sensitivity analysis excluding individuals with low physical performance at baseline (≤8 points) revealed similar results to the main analysis. This finding seems to have an important clinical implication, with D/AO associated with faster rates of physical performance decline in both early and late phases in males.

This study has several strengths. The major strength is the use of a representative national sample of community-dwelling older adults in England, which enabled us to perform analyses stratified by sex. The use of objective measures of health and physical performance (waist circumference, grip strength, and SPPB) is another strong point. Moreover, the analyses involved data from 3 waves and a long follow-up period, which enabled us to detect changes in physical performance over time. We also considered the influence of the regional redistribution of adipose tissue during the aging process, and our models were adjusted by a wide range of important covariates associated with both the exposure and outcome.

The present study also has limitations that need to be considered. First, the losses to follow-up could be a source of bias, although this type of bias is inevitable in longitudinal studies involving community-dwelling older adults. Another potential source of bias relates to the generalization of the data due to the small number of individuals in the group with dynapenic abdominal obesity. However, this fact did not affect the association with the outcome in males. Because the participants excluded from the analytical sample were poorer, had worse memory, sedentary behavior, and a greater probability of chronic diseases, the trajectories estimated for these conditions could have been underestimated. Despite the differences between the included and excluded individuals, we were able to observe a stronger decline in the physical performance of males with dynapenic abdominal obesity. The lack of information on nutrition and the history of obesity (onset and duration) constitutes another limitation. Lastly, waist circumference does not provide a direct estimate of visceral adiposity, as achieved with CT and MRI. However, it is a very useful screening tool in clinical practice.

In conclusion. dynapenic abdominal obesity is associated with a stronger decline in physical performance in older males. This finding highlights the clinical importance of including abdominal obesity and dynapenia in the evaluation of the risk of decline in physical performance, especially when these 2 conditions are found in the same patient. The identification and management of dynapenic abdominal obesity can be essential to avoiding the first signs of functional impairment in older males.

## Supplementary Material

nqac023_Supplemental_File

## Data Availability

Data from the English Longitudinal Study of Ageing (ELSA) are available from the UK Data Service for researchers who meet the criteria for access to confidential data under conditions of the End User License http://ukdataservice.ac.uk/media/455131/cd137-enduserlicence.pdf. The data can be accessed from https://www.elsa-project.ac.uk/accessing-elsa-data. Contact with the UK data service regarding access to the English Longitudinal Study of Ageing can be made through the website https://ukdataservice.ac.uk/help/.

## Notes

This work was supported by the São Paulo Research Foundation (FAPESP; grant numbers 17/26377-4 to ROM, and 18/13917-3 to TSA), the Coordination for the Improvement of Higher Education Personnel (CAPES; Finance code 001 and 88887.570076/2020-00), and the National Council of Scientific and Technological Development (CNPq; grant numbers 303981/2017-2 and 303577/2020-7). Funding for the English Longitudinal Study of Ageing is provided by the US National Institute on Aging (grant R01AG017644) and a consortium of UK governmental departments coordinated by the Economic and Social Research Council (ESRC). The funders had no involvement in the manuscript.

Supplemental Figures 1–3 and Supplemental Tables 1–4 are available from the “Supplementary data” link in the online posting of the article and from the same link in the online table of contents at https://academic.oup.com/ajcn/.

Abbreviations used: CES-D, Center for Epidemiologic Studies Depression Scale; D/AO, dynapenic/abdominal obesity; D/NAO, dynapenic/nonabdominal obesity; ELSA, English Longitudinal Study of Ageing; GLMM, generalized linear mixed model; HSE, Health Survey for England; ND/AO, nondynapenic/abdominal obesity; ND/NAO, nondynapenic/nonabdominal obesity; SPPB, Short Physical Performance Battery.
