# Supplementary material for: Combination of dynapenia and abdominal obesity affects long-term physical performance trajectories in older adults: sex differences
Source: Am J Clin Nutr. 2022 Jan 31;115(5):1290–9. doi: 10.1093/ajcn/nqac023 (PMC9071386; doi:10.1093/ajcn/nqac023)
Supplement: nqac023_Supplemental_File [file nqac023_supplemental_file.docx]

**Combination of dynapenia and abdominal obesity affects long-term physical performance trajectories in older adults: Sex differences. Máximo, RO, et al.**

**On-line Supplementary Material**

**Supplementary Table 1** – Comparison between older adults included and excluded at baseline of ELSA study (2004).

**Supplementary Table 2.** Generalized linear mixed model estimated for SPPB scores as function of abdominal obesity and dynapenia status over eight-years of follow-up in older English male and female – Including all covariates

**Supplementary Table 3.** Predictive average annual values for SPPB scores as function of abdominal and dynapenia status over eight-years of follow-up in older English male and female.

**Supplementary Table 4.** Generalized linear mixed model estimated for SPPB scores as function of abdominal obesity and dynapenia status over eight-years of follow-up in older English male and female – Sensitivity analysis excluding individuals with low physical performance at baseline (≤ 8 points).

**Supplementary Figure 1.** Participant Flowchart.

**Supplementary Figure 2.** Trajectories of performance on SPPB for male according to abdominal obesity and dynapenia status – ELSA Study 2004-2012 – Sensitivity analysis excluding individuals with low physical performance at baseline (≤ 8 points).

**Supplementary Figure 3.** Trajectories of performance on SPPB for female according to abdominal obesity and dynapenia status – ELSA Study 2004-2012 – Sensitivity analysis excluding individuals with low physical performance at baseline (≤ 8 points).

**Supplementary Table 1** – Comparison between older adults included and excluded at baseline of ELSA study (2004).

|  | **Included** | **Excluded** |
| --- | --- | --- |
| Sex, female | 54.1***** | 57.9***** |
| Age, years | 70.0 ± 7.1***** | 73.7 ± 8.6***** |
| 60 - 69 years | 52.7***** | 36.1***** |
| 70 - 79 years | 35.8 | 34.7 |
| 80 y or more | 11.5***** | 29.2***** |
| Marital status (not married), (%) | 32.9***** | 46.5***** |
| Total household wealth in quintiles, (%) |  |  |
| 1^st^ (highest) | 23.7***** | 13.8***** |
| 2^nd^ | 22.3* | 14.9***** |
| 3^rd^ | 21.1 | 18.2 |
| 4^th^ | 18.2***** | 22.8***** |
| 5^th^ (lowest) | 13.6* | 29.5***** |
| Not reported, (%) | 1.1 | 0.8 |
| Level of education, (%) |  |  |
| Higher qualification | 23.5* | 15.6***** |
| Level lower than “A level” or equivalent | 21.6***** | 15.4***** |
| Level lower than “O level” or equivalent | 54.9* | 69.0***** |
| Smoking, (%) |  |  |
| Non-smoker | 37.3* | 33.5***** |
| Ex-smoker | 51.3 | 51.2 |
| Smoker | 11.4* | 15.3***** |
| Alcohol intake, (%) |  |  |
| Non-drinker or rare drinker | 17.1***** | 23.4***** |
| Frequent drinker | 42.2***** | 28.4***** |
| Daily drinker | 32.5***** | 21.9***** |
| Did not answer | 8.2***** | 26.3***** |
| Sedentary behavior, (%) | 2.6***** | 18.2***** |
| Hypertension, (%) | 46.0***** | 53.2***** |
| Diabetes, (%) | 8.2***** | 13.5***** |
| Cancer, (%) | 9.1 | 9.2 |
| Lung disease, (%) | 17.5***** | 21.0***** |
| Heart disease, (%) | 23.6***** | 32.6***** |
| Stroke, (%) | 4.2***** | 11.4***** |
| Osteoarthritis, (%) | 36.6***** | 52.6***** |
| Osteoporosis, (%) | 7.0***** | 11.7***** |
| Joint pain, (%) | 23.7* | 39.0***** |
| Number of falls in previous 12 months, (mean ± SD) | 0.6 ± 1.9* | 1.9 ± 14.5***** |
| Depressive symptoms, (%) | 11.0***** | 23.0***** |
| Memory score, points (mean ± SD) | 9.7 ± 3.4***** | 8.2 ± 3.7***** |
| Grip strength, kg (mean ± SD) | 30.7 ± 10.5***** | 25.6 ± 11.1***** |
| Waist circumference, cm (mean ± SD) | 95.1 ± 12.4***** | 97.7 ± 14.1***** |
| Height, m (mean ± SD) | 1.6 ± 0.9 | 1.6 ± 0.9 |
| BMI ≥30 kg/m^2^, (%) | 26.2* | 34.8***** |

Chi-squared test performed for categorical variables; analysis of variance (ANOVA) and Tukey’s post hoc test performed for continuous variables to evaluate differences in baseline characteristics between included individuals and those excluded due to missing data on SPPB, grip strength, waist circumference or other covariates. Data expressed as percentage, mean and standard deviation (SD). Statistical significance: p-value <0.05. Included, n = 3,875; Excluded, n = 2,308.

**Supplementary Table 2.** Generalized linear mixed model estimated for SPPB scores as function of abdominal obesity and dynapenia status over eight-years of follow-up in older English male and female – including all covariates.

|  | **Male** | | **Female** | |
| --- | --- | --- | --- | --- |
|  | **Estimated Parameters ß (95% CI)** | | **Estimated Parameters ß (95% CI)** | |
| **Intercept Main effect** |  |  |  |  |
| Age |  |  |  |  |
| 70 - 79 years | -0.71 | (-0.86, -0.56)****** | -0.82 | (-0.98, -0.66)****** |
| 80 y or more | -1.59 | (-1.82, -1.35)****** | -1.82 | (-2.07, -1.57)****** |
| Total household wealth in quintiles |  |  |  |  |
| 2^nd^ | 0.05 | (-0.14, 0.24) | -0.00 | (-0.22, 0.21) |
| 3^rd^ | -0.10 | (-0.30, 0.11) | -0.21 | (-0.43, 0.01) |
| 4^th^ | -0.22 | (-0.45, 0.00) | -0.47 | (-0.70, -0.24)****** |
| 5^th^ (lowest) | -0.24 | (-0.50, 0.02) | -0.53 | (-0.79, -0.27)****** |
| Level of education |  |  |  |  |
| Level lower than “A level” or equivalent | -0.05 | (-0.24, 0.14) | -0.02 | (-0.24, 020) |
| Level lower than “O level” or equivalent | -0.25 | (-0.42, -0.07)***** | -0.17 | (-0.37, 0.04) |
| Marital status (not married) | -0.17 | (-0.34, -0.00)***** | -0.08 | (-0.23, 0.08) |
| Smoking |  |  |  |  |
| Ex-smoker | 0.03 | (-0.13, 0.18) | 0.02 | (-0.13, 0.17) |
| Smoker | -0.23 | (-0.47, 0.01) | 0.05 | (-0.19, 0.30) |
| Sedentary behavior | -1.05 | (-1.48, -0.63)****** | -1.06 | (-1.47, -0.64)****** |
| Hypertension | 0.09 | (-0.05, 0.23) | -0.08 | (-0.22, 0.07) |
| Diabetes | -0.20 | (-0.43, 0.02) | -0.43 | (-0.72, -0.15)***** |
| Lung disease | -0.17 | (-0.35, 0.02) | -0.22 | (-0.40, -0.03)***** |
| Heart disease | -0.04 | (-0.20, 0.11) | -0.11 | (-0.28, 0.07) |
| Stroke | -0.48 | (-0.80, -0.16)***** | -0.66 | (-1.03, -0.30)****** |
| Osteoarthritis | -0.16 | (-0.32, -0.00)***** | -0.29 | (-0.45, -0.14)****** |
| Osteoporosis | -0.12 | (-0.64, 0.40) | -0.35 | (-0.57, -0.13)***** |
| Number of falls in previous 12 months | -0.04 | (-0.08, -0.00)***** | -0.05 | (-0.09, -0.01) |
| Joint pain | -0.31 | (-0.49, -0.13)***** | -0.28 | (-0.45, -0.10)***** |
| Depressive symptoms | -0.30 | (-0.56, -0.03)***** | -0.27 | (-0.48, -0.07)***** |
| Memory score | 0.05 | (0.03, 0.07)****** | 0.05 | (0.03, 0.07)****** |
| Change weight |  |  |  |  |
| Lose weight | -0.51 | (-1.03, 0.01) | -0.94 | (-1.39, -0.48)****** |
| Gain weight | -0.73 | (-1.33, -0.14)***** | -0.71 | (-1.20, -0.23)***** |
| **Slope Interaction effect** |  |  |  |  |
| Time x Age |  |  |  |  |
| 70 - 79 years | 0.09 | (0.05, 0.14)****** | 0.05 | (0.00, 0.09)***** |
| 80 y or more | 0.07 | (0.01, 0.13)***** | -0.01 | (-0.07, 0.05) |
| Time x Total household in quintiles |  |  |  |  |
| 2^nd^ | -0.06 | (-0.11, -0.01)***** | -0.05 | (-0.10, 0.01) |
| 3^rd^ | 0.00 | (-0.05, 0.06) | -0.00 | (-0.06, 0.05) |
| 4^th^ | -0.00 | (-0.07, 0.06) | -0.03 | (-0.09, 0.03) |
| 5^th^ (lowest) | -0.11 | (-0.19, -0.04)***** | -0.06 | (-0.13, 0.00) |
| Time x Level of education | -0.03 | (-0.07, 0.02) | -0.03 | (-0.07, 0.01) |
| Level lower than “A level” or equivalent | 0.00 | (-0.05, 0.05) | 0.05 | (-0.01, 0.10) |
| Level lower than “O level” or equivalent | -0.03 | (-0.08, 0.02) | 0.02 | (-0.02, 0.07) |
| Time x Marital status (not married) |  |  |  |  |
| Time x Smoking |  |  |  |  |
| Ex-smoker | 0.03 | (-0.01, 0.08) | -0.01 | (-0.05, 0.02) |
| Smoker | 0.05 | (-0.03, 0.12) | -0.02 | (-0.09, 0.05) |
| Time x Sedentary behavior | -0.20 | (-0.30, -0.10)****** | -0.08 | (-0.17, 0.02) |
| Time x Hypertension | -0.02 | (-0.06, 0.02) | -0.04 | (-0.07, 0.00) |
| Time x Diabetes | 0.03 | (-0.03, 0.09) | -0.06 | (-0.13, -0.00)***** |
| Time x Lung disease | -0.03 | (-0.09, 0.02) | -0.02 | (-0.06, 0.03) |
| Time x Heart disease | -0.02 | (-0.06, 0.02) | -0.02 | (-0.07, 0.02) |
| Time x Stroke | 0.01 | (-0.07, 0.08) | -0.01 | (-0.09, 0.07) |
| Time x Osteoarthritis | 0.01 | (-0.04, 0.05) | -0.00 | (-0.04, 0.03) |
| Time x Osteoporosis | -0.11 | (-0.23, 0.02) | -0.03 | (-0.08, 0.02) |
| Time x Number of falls in previous 12 months | 0.00 | (-0.00, 0.01) | 0.00 | (-0.01, 0.00) |
| Time x Joint pain | -0.03 | (-0.08, 0.02) | -0.06 | (-0.10, -0.02)***** |
| Time x Depressive symptoms | -0.05 | (-0.13, 0.02) | -0.07 | (-0.49, -0.07)* |
| Time x Memory score | 0.01 | (0.01, 0.02)****** | 0.01 | (0.01, 0.02)****** |
| Time x Change weight |  |  |  |  |
| Lose weight | 0.01 | (-0.07, 0.10) | 0.09 | (0.01, 0.16)***** |
| Gain weight | 0.14 | (0.04, 0.24)***** | 0.10 | (0.02, 0.19)***** |

Generalized linear mixed models performed estimated beta coefficients (ß) and 95% confidence intervals (CI) for SPPB scores as function of abdominal obesity and dynapenia status in older adults. Statistical significance: ***** p < 0.05; ****** p < 0.001. Male, n = 1,780; female, n = 2,095.

**Supplementary Table 3.** Predictive average annual values for SPPB scores as function of abdominal and dynapenia status over eight-years of follow-up in older English male and female.

| **Male** | | | | | | | | | |
| --- | --- | --- | --- | --- | --- | --- | --- | --- | --- |
|  | **ND/NAO** | | **ND/AO** | | **D/NAO** | | **D/AO** | | |
|  | Predictive | 95% CI | Predictive | 95% CI | Predictive | 95% CI | Predictive | 95% CI | |
| Baseline | 10.50 | 10.39, 10.62 | 10.23 | 10.12, 10.36 | 9.33 | 8.97, 9.71 | 9.39 | 8.94, 9.85 | |
| 1 year | 10.44 | 10.35, 10.54 | 10.15 | 10.05, 10.26 | 9.26 | 8.95, 9.58 | 9.22 | 8.83, 9.61 | |
| 2 years | 10.38 | 10.29, 10.47 | 10.06 | 9.97, 10.16 | 9.20 | 8.91, 9.48 | 9.05 | 8.71, 9.39 | |
| 3 years | 10.32 | 10.22, 10.41 | 9.98 | 9.88, 10.08 | 9.12 | 8.85, 9.40 | 8.88 | 8.57, 9.19 | |
| 4 years | 10.25 | 10.15, 10.36 | 9.89 | 9.78, 10.01 | 9.05 | 8.76, 9.34 | 8.71 | 8.39, 9.02 | |
| 5 years | 10.19 | 10.07, 10.32 | 9.80 | 9.67, 9.94 | 8.98 | 8.65, 9.31 | 8.53 | 8.19, 8.88 | |
| 6 years | 10.13 | 9.98, 10.28 | 9.72 | 9.56, 9.88 | 8.91 | 8.53, 9.29 | 8.36 | 7.97, 8.75 | |
| 7 years | 10.07 | 9.89, 10.24 | 9.63 | 9.45, 9.82 | 8.84 | 8.39, 929 | 8.19 | 7.73, 8.65 | |
| 8 years | 10.01 | 9.80, 10.20 | 9.54 | 9.33, 9.76 | 8.77 | 8.25, 9.29 | 8.02 | 7.49, 8.55 | |
| **Female** | | | | | | | | |  |
|  | **ND/NAO** | | **ND/AO** | | **D/NAO** | | **D/AO** | | |
|  | Predictive | 95% CI | Predictive | 95% CI | Predictive | 95% CI | Predictive | 95% CI | |
| Baseline | 10.02 | 9.90, 10.16 | 9.65 | 9.54, 9.78 | 9.19 | 8.84, 9.56 | 8.63 | 8.28, 8.99 | |
| 1 year | 9.97 | 9.86, 10.09 | 9.59 | 9.49, 9.70 | 9.13 | 8.83, 9.46 | 8.59 | 8.29, 8.90 | |
| 2 years | 9.92 | 9.81, 10.03 | 9.53 | 9.43, 9.63 | 9.10 | 8.82, 9.38 | 8.56 | 8.29, 8.82 | |
| 3 years | 9.86 | 9.75, 9.97 | 9.46 | 9.37, 9.56 | 9.05 | 8.78, 9.32 | 8.51 | 8.27, 8.76 | |
| 4 years | 9.81 | 9.69, 9.93 | 9.40 | 9.29, 9.50 | 9.01 | 8.73, 9.28 | 8.47 | 8.23, 8.72 | |
| 5 years | 9.75 | 9.61, 9.90 | 9.33 | 9.21, 9.45 | 8.96 | 8.65, 9.27 | 8.43 | 8.17, 8.70 | |
| 6 years | 9.70 | 9.53, 9.86 | 9.27 | 9.13, 9.40 | 8.91 | 8.56, 9.26 | 8.39 | 8.09, 8.70 | |
| 7 years | 9.64 | 9.45, 9.84 | 9.20 | 9.04, 9.36 | 8.86 | 8.46, 9.27 | 8.35 | 8.00, 8.70 | |
| 8 years | 9.59 | 9.37, 9.81 | 9.14 | 8.96, 9.32 | 8.82 | 8.35, 9.28 | 8.31 | 7.91, 8.72 | |

Margins statistics calculated from predictions of previously fitted model at fixed values for some covariates and averaging. Male, n = 1,780; Female, n = 2,095. Abbreviations: D/AO, dynapenic/abdominal obesity; D/NAO, dynapenic/non-abdominal obesity; ND/AO, non-dynapenic/abdominal obesity; ND/NAO, non-dynapenic/non-abdominal obesity.

**Supplementary Table 4.** Generalized linear mixed model estimated for SPPB scores as function of abdominal obesity and dynapenia status over eight-years of follow-up in older English male and female – Sensitivity analysis excluding individuals with low physical performance at baseline (≤ 8 points).

|  | **Male** | | **Female** | |
| --- | --- | --- | --- | --- |
|  | **Estimated Parameters ß (95% CI)** | | **Estimated Parameters ß (95% CI)** | |
| Time, Years | -0.22 | (-0.31, -0.12)****** | -0.15 | (-0.24, -0.05)***** |
| **Intercept Main effect** |  |  |  |  |
| ND/NAO | 10.83† |  | 10.62† |  |
| ND/AO | -0.18 | (-0.31, -0.06)***** | -0.30 | (-0.42, -0.17)****** |
| D/NAO | -0.61 | (-1.02, -0.20)***** | -0.32 | (-0.71, 0.06) |
| D/AO | -0.61 | (-1.12, -0.11)***** | -0.69 | (-1.09, -0.29)****** |
| **Slope Interaction effect** |  |  |  |  |
| Time x ND/NAO | 10.74† |  | 10.53† |  |
| Time x ND/AO | -0.04 | (-0.08, 0.00) | -0.03 | (-0.07, 0.01) |
| Time x D/NAO | -0.09 | (-0.18, 0.01) | -0.06 | (-0.15, 0.03) |
| Time x D/AO | -0.16 | (-0.27, -0.05)***** | -0.04 | (-0.13, 0.04) |

Generalized linear mixed models performed to estimate beta coefficients (ß) and 95% confidence intervals (CI) for SPPB scores as function of abdominal obesity and dynapenia status in older adults excluding individuals with low physical performance at baseline (≤ 8 points). Model adjusted for age, total household wealth, years of schooling, marital status, smoking status, sedentary behavior, diabetes, lung disease, heart disease, stroke, osteoarthritis, osteoporosis, number of falls, joint pain, depressive symptoms, mean memory score and change in weight. Intercept represents differences in mean SPPB score between ND/AO, D/NAO, and D/AO and reference group (ND/NAO) at baseline. Time represents SPPB performance decline in reference group. Slope represents estimated changes in SPPB scores per unit of time between group in question and reference. Significantly different from ND/NAO, ***** p < 0.05; ****** p < 0.001. † indicates margins to reference group. Male, n = 1,590; female, n = 1,744. Abbreviations: D/AO, dynapenic/abdominal obesity; D/NAO, dynapenic/non-abdominal obesity; ND/AO, non-dynapenic/abdominal obesity; ND/NAO, non-dynapenic/non-abdominal obesity.

**Supplementary Figure 1.** Participant Flowchart.


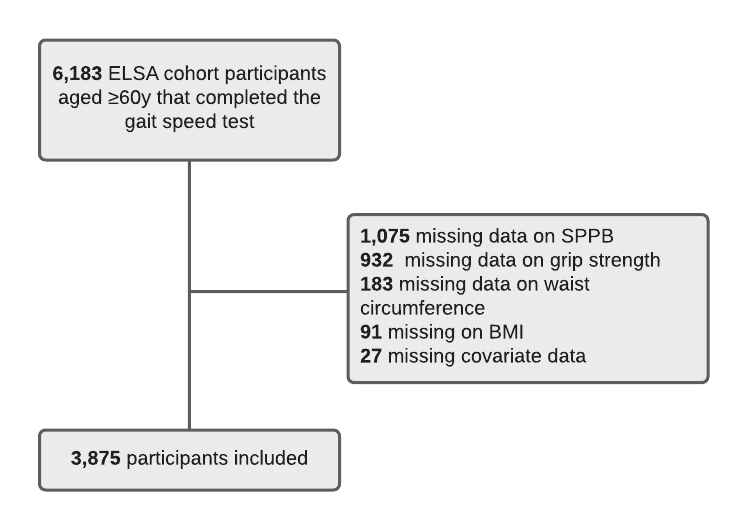


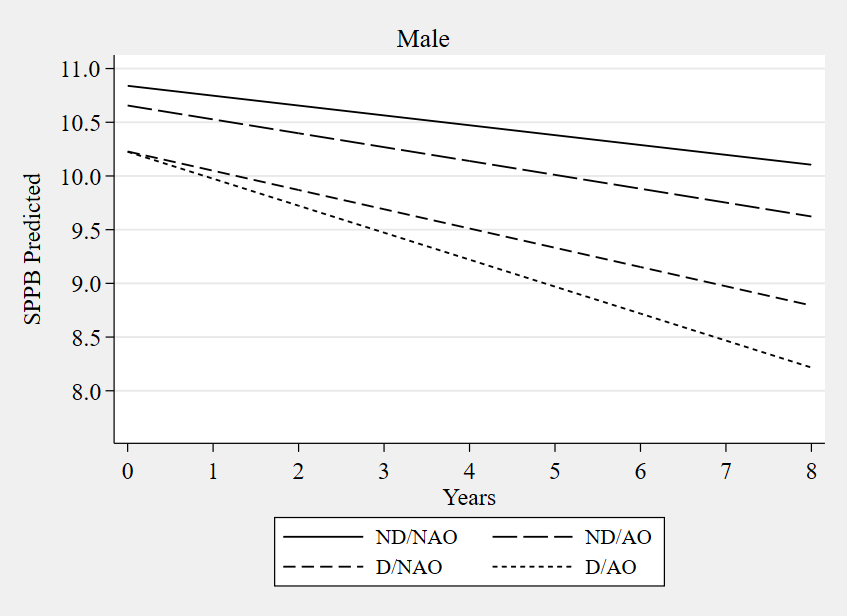


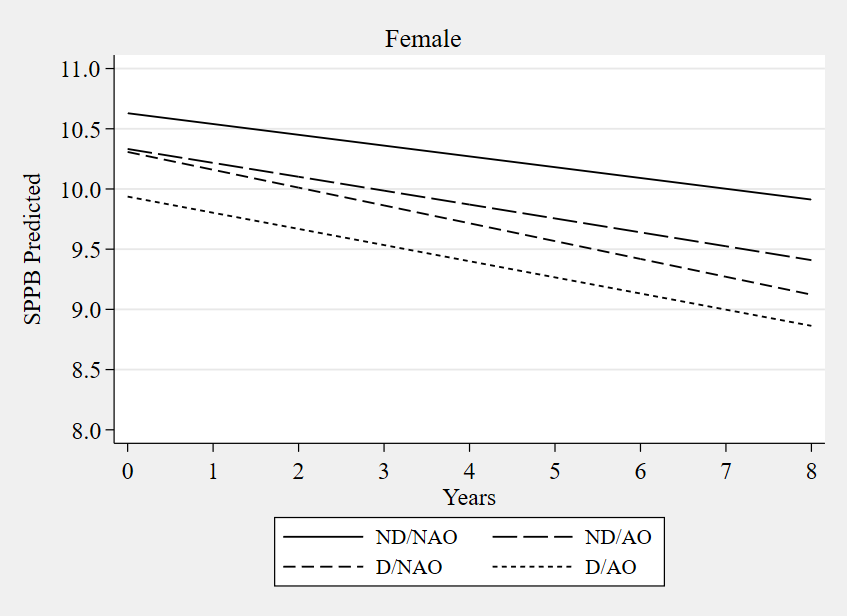


**Supplementary Figure 2.** Trajectories of performance on SPPB for male according to abdominal obesity and dynapenia status – ELSA Study 2004-2012 – Sensitivity analysis excluding individuals with low physical performance at baseline (≤ 8 points). Predictions for 60 years of age, male, total household wealth = 1^st^ quintile, higher qualification, married, non-smokers, active, without hypertension, without diabetes, without lung disease, without heart disease, without stroke, without osteoarthritis, without osteoporosis, without falls, without joint pain, *CESD* <4 points, mean memory score = 20 and stable weight. Over the eight-year follow-up, only males with D/AO had a faster rate of decline in the SPPB performance compared to males in the ND/NAO group (-0.16 points per year; 95% CI: -0.27, -0.05; p <0.01). n = 1,590. Abbreviations: D/AO, dynapenic/abdominal obesity; D/NAO, dynapenic/non-abdominal obesity; ND/AO, non-dynapenic/abdominal obesity; ND/NAO, non-dynapenic/non-abdominal obesity. Figure created with STATA 14 (StataCorp. 2015. *Stata Statistical Software: Release 14.* College Station, TX: StataCorp LP) (1).

**Supplementary Figure 3.** Trajectories of performance on SPPB for female according to abdominal obesity and dynapenia status – ELSA Study 2004-2012 – Sensitivity analysis excluding individuals with low physical performance at baseline (≤ 8 points). Predictions for 60 years of age, female, total household wealth = 1^st^ quintile, higher qualification, married, non-smokers, active, without hypertension, without diabetes, without lung disease, without heart disease, without stroke, without osteoarthritis, without osteoporosis, without falls, without joint pain, *CESD* <4 points, mean memory score = 20 and stable weight. Females with D/AO underwent no significant decline in the performance on the SPPB over time (-0.04 points per year; 95% CI: -0.13, 0.04; p =0.32). n = 1,744. Abbreviations: D/AO, dynapenic/abdominal obesity; D/NAO, dynapenic/non-abdominal obesity; ND/AO, non-dynapenic/abdominal obesity; ND/NAO, non-dynapenic/non-abdominal obesity. Figure created with STATA 14 (StataCorp. 2015. *Stata Statistical Software: Release 14.* College Station, TX: StataCorp LP) (1).

**Reference**

1. StataCorp. 2015. Stata Statistical Software: Release 14. College Station, TX: StataCorp LP.
